# Demand for forest ecosystem services: a comparison study in selected areas in the Czech Republic and China

**DOI:** 10.1007/s10342-022-01478-0

**Published:** 2022-07-23

**Authors:** Miroslava Hochmalová, Ratna Chrismiari Purwestri, Jian Yongfeng, Vilém Jarský, Marcel Riedl, Dian Yuanyong, Miroslav Hájek

**Affiliations:** 1 Faculty of Forestry and Wood Sciences, Czech University of Life Sciences Prague, Kamýcká 129, 6–Suchdol, 16500 Prague, Czech Republic; 2 College of Horticulture and Forestry Science, Huazhong Agricultural University, Wuhan, 4300070 Hubei People’s Republic of China

**Keywords:** Demand for ecosystem services, Forest ecosystem services, Forest visitor’s expectations, Cross-cultural analyses

## Abstract

Ecosystem services are investigated from many perspectives, but there are very few studies comparing the perception of forest and demand for forest ecosystem services (FES) in a cross-cultural analysis. This study aims to map the demand for FES and find out the forest perception of forest visitors in both Czech and Chinese societies. Data were collected by structured questionnaire among three different groups of respondents (*n* = 847) in six forest areas. The questions were focused on the demand for FES, expectations from the forest, preference for the visual form of the forest, and the willingness of forest visitors. Analysis demonstrates that the demand for some FES is related to geographical and cultural conditions. The results indicated that provisioning and regulation services are perceived as more important than cultural services. The differences by country were obvious in the cultural and provisioning services: Chinese demand more relaxing and meditation activities, whereas Czech demand mushroom picking. A significant outcome is a high demand of Chinese respondents for recreational facilities. Tree planting was rated as one of the most popular voluntary activity across the whole sample. Meanwhile, some findings point to an increasing pressure on forest ecosystems and their protection, which emerge due to the strong demand for recreational facilities. According to the findings, active involvement of forest visitors in various activities is recommended so that their appreciation of FES will constantly increase and to take into account the profile of visitors and incorporate them in forest management and planning in order to meet societal demand.

## Introduction

The continuous growth and worldwide expansion of the human population has caused widespread degradation of natural ecosystems (Millennium Ecosystem Assessment 2005; Tilman and Lehman 2001; Vitousek 1994). The fact that people do not appreciate the environmental, economic, and social values provided by nature, and do not realize the impact that lack of interest on ecosystems creates (Loomes and O’Neill 2000; Balmford et al. 2002; Foley et al. 2005), gives rise to well-founded concern about the depletion of natural resources and the ability of nature to continue providing ecosystem services (ES) (Costanza et al. 1997). The supply of forest ES is linked to the products and services provided by forest ecosystems for human well-being. The demand for forest ES is the human consumption of these products and services (Sukhdev et al. 2010; Braat and de Groot 2012). The provision of ES depends on the capacity of a particular area to provide locally determined ES and goods for satisfying human needs within a given period. To achieve sustainable development of ES, it is essential to promote the ecological interest and information within the society, and to care for natural ecosystems in such a way as to preserve them for future generations (Burkhard et al. 2012; Angelstam et al. 2013). In response to deteriorating conditions of natural ecosystems, the Millennium Ecosystem Assessment was issued in 2005 a milestone in the EC issue. Thanks to this document, the concept of ES was unified and further divided into three basic categories: provisioning services (nutrients, materials, energy), regulation services (waste regulation, flow of physical and biotic environment), and cultural services (symbolic, educational, experimental) (Millennium Ecosystem Assessment 2005). The study carried out by Frélichová et al. (2014) identifies with benefit or transfer approach 17 ES within the Czech Republic, the most valuable in relation to forests are timber provision, aesthetic value, erosion regulation, climate regulation, and also recreation services. The value of forest ecosystem services reaches almost 90 thousand EUR/ha, i.e., 18% of all ES.

ES demand encompasses all of ES and goods used and consumed in a particular area within the same time period (Burkhard et al. 2011). To achieve sustainable management are necessary quantification and research of the relationship of the ES supply and demand (Khosravi Mashizi and Sharafatmandrad 2021). Various mapping and assessing approaches of ecosystem services have been proposed. However, all of them are crucial for understanding how ecosystems contribute to human wellbeing and to support policies related to natural resources (Burkhard and Maes 2017). Spatial assessment tools are being developed to evaluate the impact of spatial planning on a wide range of ecosystems (Nelson et al. 2009; Kandziora et al. 2013; Bagstad et al. 2014). However, spatial maps can be really useful but also short-sighted and can mask underlying processes (Hauck et al. 2013). Many studies are carried out in a top-down way by map and modeling ecosystem services in large-scale processes and interactions for estimating consequences for stakeholders. However, few studies deal with the bottom-up viewpoint manner, thus stakeholders are placed as a focal target group (Müller et al. 2010). Evidence-based ranking of potential demand of ecosystem services can be determined through questionnaires, but also involves basic needs (Burkhard and Maes 2017); García-Nieto et al. (2013) assessed the perception of ecosystem services by face-to-face method as an indicator of how respondents valued and demand them. The main findings were that the most demanded ecosystem service was nature tourism, followed by timber, erosion control, recreational hunting, mushroom picking, and beekeeping. Differences were found between the different types of respondents. Moreover, the outcomes of the mapping can be a valuable communication and decision-making tool (Crossman et al. 2013; García-Nieto et al. 2013). However, for their practical use in forestry and conservation policy, supportive studies are needed as a facilitator for policy decisions and meeting the goals of the MEA (Ash et al. 2010; Seppelt et al. 2011). To meet the MEA objectives and sustainably use the forest natural capital, it is necessary to strike a balance between ES production and consumption (Ala-Hulkko et al. 2019). According to Syrbe and Grunewald (2017), it is necessary to thoroughly analyze the demand for the ES to prevent the misuse of primary resources by large market players. A great deal of research has been devoted to forest attendance issue or to motivation of forest visits, which gaining much attention especially due to COVID-19 pandemic (Derks et al. 2020; Venter et al. 2020; Jarský et al. 2022). Some studies analyze the perception of urban parks or urban forests and their ES. Korean study of (Jang-Hwan et al. 2020) explored that most appreciated in urban parks are regulation services. In Slovenian study of Nastran et al. (2022) indicate that stakeholders in the urban forest demand most regulation and cultural services. In addition, researchers and youngsters consider ES as more important than others. However, the most valued ES in a Belgian study carried out by Buchel and Frantzeskaki (2015) were aesthetic and cultural. The British study of Collins et al. (2019) explored public perception of ES provide by urban park trees, the provision and regulation were most appreciated. In Chengdu, China, regulation service was considered by far the most important (Swapan et al. 2017). Interesting contribution to conservative education bring Torkar and Krašovec (2019), their findings show that students with better ecology knowledge placed more importance on regulating services. Cross-cultural differences in forest preferences and attitudes toward forests have rarely been studied. Arnberger et al. (2010) compared the preferences of visitors to urban forests in Austria and Japan for social conditions in the outdoor environment. Differences were identified in higher demands for social stimulation for Japanese respondents. In a cross-cultural comparison study of Switzerland and China Lindemann-Matthies et al. (2013) found that all participants highly valued the ecosystem services provided by forests, especially the regulating and supporting ones. Another cross-cultural comparative study of urban green infrastructure perceiving among urban tourists was carried out in eight European countries (Terkenli et al. 2020). The respondents were influenced in some preferences by the in-grained activities from their countries of origin. Although the regulation services seem most appreciated, the above outcomes also indicate that cultural differences as well as groups of users, but also their characteristics, and drivers for visiting the forest may influence perceptions of ES.

The aim of this paper is to map the societal demand of FES, the attitudes, and perception of the forest in study areas in the Czech Republic and China, and to provide a current overview of social attitudes on the importance of ES. The data collected via questionnaire can contribute to a better cross-cultural understanding, and in identifying the weak and strong points, primarily in the societal demand of forest recreation. The results of the study can be one of the sources to balance the supply and demand while reflecting the potential opportunities and challenges for forestry management and planning.

## Material and methods

### Study areas

#### Czech Republic

The Czech Republic is a Central European country; forests cover 34.1% of the total land area. In the forest composition, conifers (71%) predominate, especially Norway spruce (Picea abies). The share of broadleaves is 27% (Ministry of Agriculture, 2019). The climate is temperate, with an average annual precipitation of 686 mm and temperature of 7.9 °C (Czech Hydrometeorological Institute 2022).

A wide network of protected areas with natural attractions is intertwined by hiking trails as outdoor recreation in natural areas has a long tradition there. Moreover, access to the forest is free of charge. People mostly visit the forest for a walk or to enjoy the outdoors. The average visitation per person in 2019 was 22.9 visits annually, and the trend has been growing in the three last years (Ministry of Agriculture 2019; Šodková et al. 2020). The Czechs traditionally gather wild plants, mushrooms, and edible berries. Wild mushroom gathering has a long tradition in the cultural history of Central and Eastern Europe (Riedl et al. 2020; Seeland and Staniszewski 2007; Šišák et al. 2016; Šišák and Pulkrab 2009). Mushrooms were once among primary sources of daily food, nowadays it is mainly a recreational activity, while some people also sell mushrooms in local markets (Martínez de Aragón et al. 2011).

**Fig. 1:**
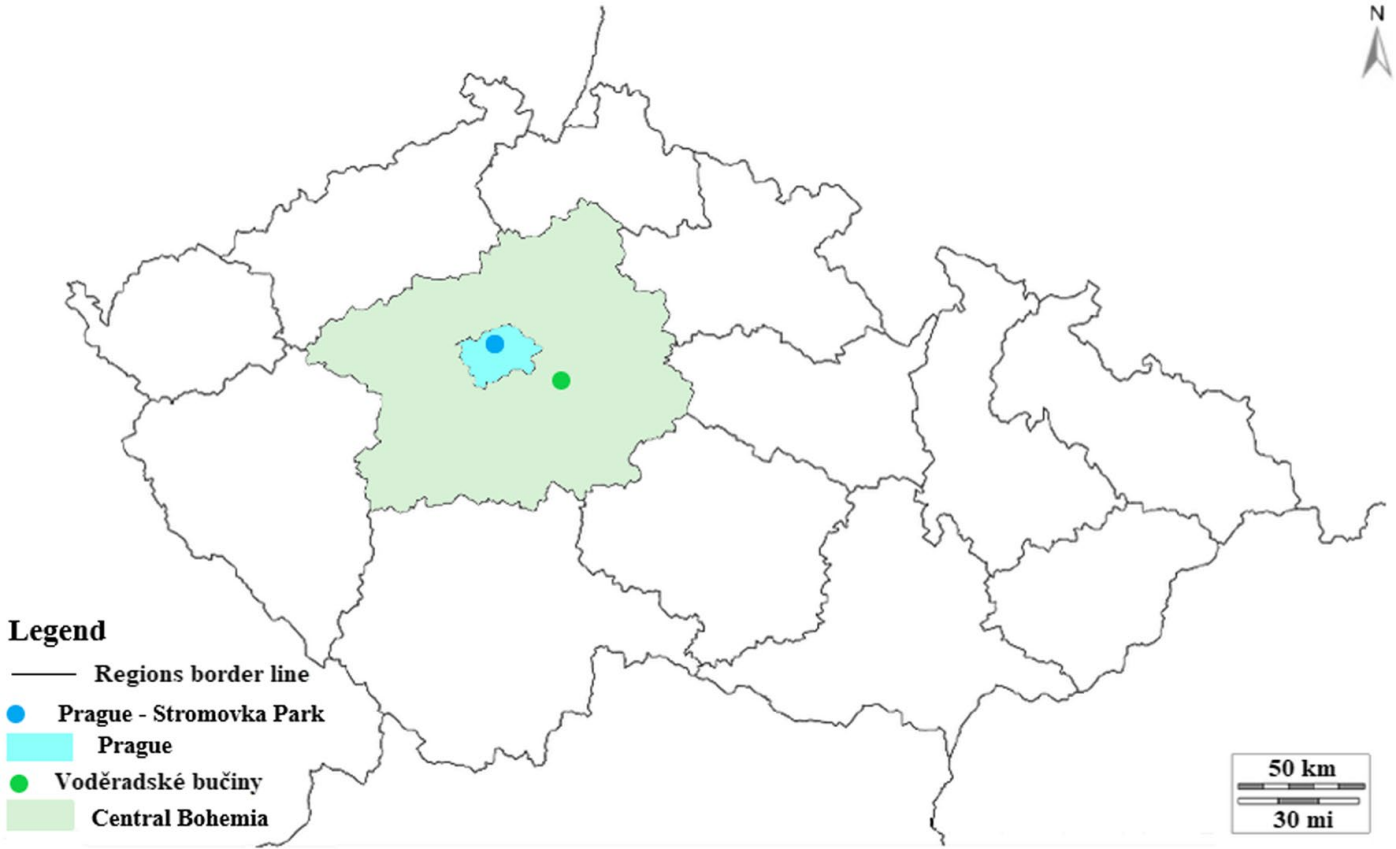
Map of the study areas in the Czech Republic

The first study area where a group of tourists was interviewed was the Voděradské bučiny National Nature Reserve. This location is popular for its high recreation value because it is easily accessible and there is a tourist trail through a beech forest with wells, streams, and ponds. A group of urban dwellers were interviewed in Stromovka Park, one of the oldest city parks in Prague, and also very valuable for its urban landscape aesthetics. Many visitors relax, play sports, or just walk there People’s Republic of China (Fig. 1).

#### Hubei-Wuhan

**Fig. 2:**
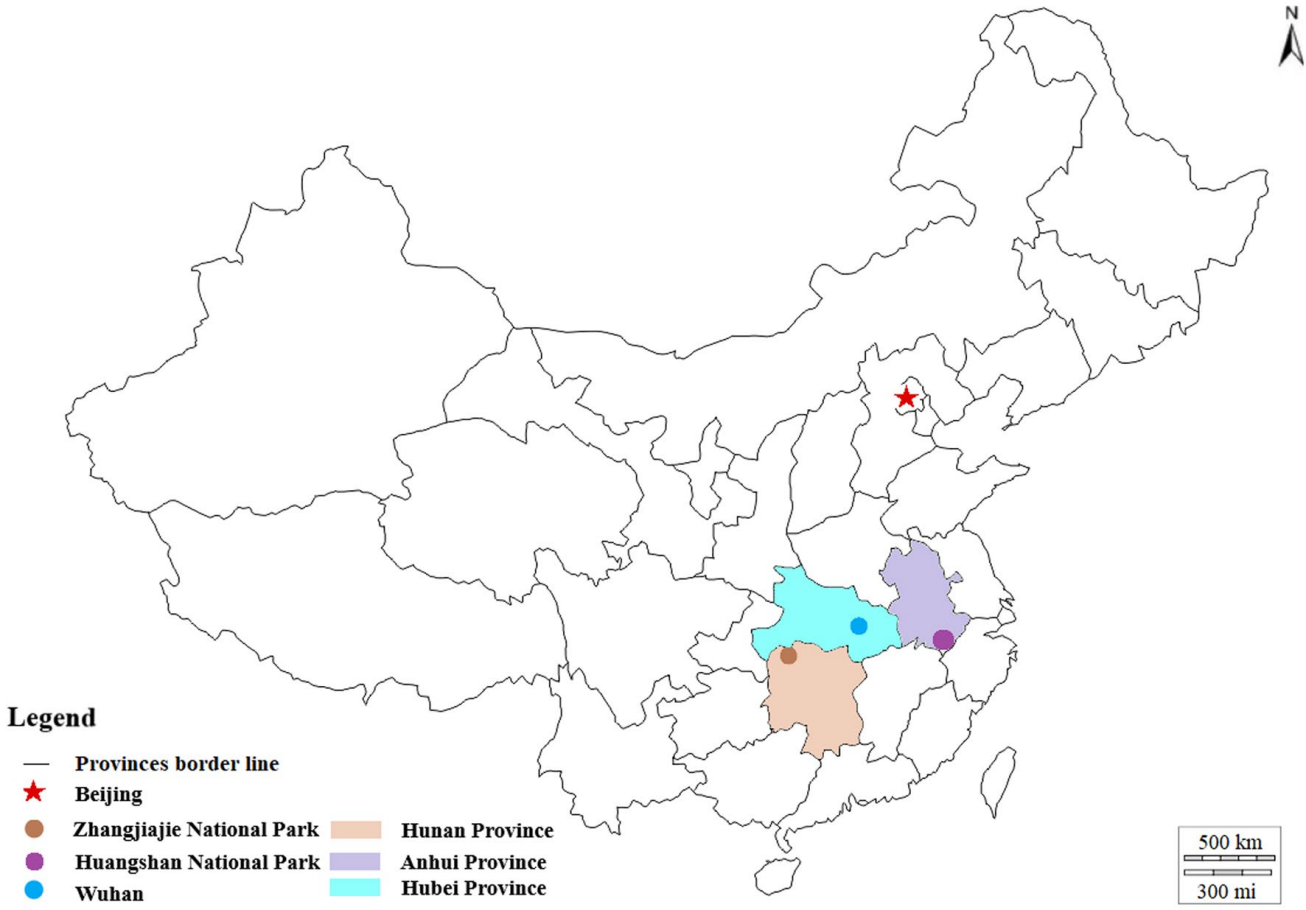
Map of the study areas in the Republic of China

Wuhan is the capital city of Hubei Province located in central China (Fig. 2). The city is divided by the merging of the Yangtze and Han rivers into three parts. The population has reached 11.2 million inhabitants in 2019 (Wuhan Bureau of Statistics 2020). The climate is humid subtropical with hot and rainy summers, and cool and dry winters with an annual temperature of 16.3 °C (Huang et al. 2021). The survey was administered in the East Lake Scenic Area and the Lion Mountain Park in Wuhan. Both parks are dominated by evergreen and deciduous broadleaved mixed forests (Zhu et al. 2019).

#### Anhui – Huangshan

The Huangshan National Park is located in the humid monsoon climate zone in Southeast China’s Anhui province (Fig. 2), a predominately subtropical evergreen broadleaved forest. The area of the National Park is 330.3 km^2^ of which 94.9% are forest ecosystems (Yu et al. 2020). Mount Huangshan is listed in the UNESCO Natural and Cultural Heritage Site and Global Geopark list. The National Park is under the protection of the laws of China, Forestry law, and the Law on the Protection of Wildlife. The location is rich in flora, especially mosses, lichens, and ferns. In addition, there are also more than 300 species of fauna, including endangered genera. In recent decades, the National Park has been facing high pressure in tourism, with 2.74 million visitors per year. Visitors can move on 30 mostly paved hiking trails, which results in a growing amount of waste that the park management has to deal with. The trails are unique with a number of stone steps (Hu et al. 2018).

#### Hunan – Zhangjiajie

The Zhangjiajie National Forest Park is a part of the Wuliang scenic area, Northwestern Hunan Province (Fig. 2). In 1982, it became the first forest National Park in China and is part of the scenic area included in the UNESCO World Natural Heritage list. The main characteristics of Zhangjiajie are more than 3000 sandstone rock peaks and pillars on 126 km^2^ (Unesco 2022). The forest is rich in plant species, 106 families, and 850 species of woody plants with a coverage rate of 97.7%. In the primeval forest, there are precious ancient tree species such as *Davidia involucrata, Ginkgo biloba*, or various coniferous in *Taxus* genus. The park belongs to the mid-subtropical climate zone with an average annual temperature of 16.5 °C (Xie et al. 2005). The park is conveniently accessible from the city of Zhangjiajie. In total, there are 58 km of hiking trails in the scenic area, ropeways, and tourist vehicle trails (Tourism Publicity and Promotion Management Office 2021).

### Sampling design

The survey was carried out in 2019–2020 in China and in the Czech Republic. Altogether, three groups of respondents (*n* = 846) in each country were interviewed. The second part of the research in 2020 was influenced by the Covid-19 pandemic situation. Due to the limitation of free movement in the Czech Republic, it was impossible to interview people in the forest. Therefore, the research continued after the relaxation of pandemic restrictions in summer 2020. Similar approach was conducted by Lindemann-Matthies et al. (2013), they interviewed Czech and Swiss respondents divided into two study groups, an urban and a rural.

All groups of respondents are dependent directly or indirectly on ES for their wellbeing. However, environmental, economic, and social differences can influence people's relationship with nature. The prerequisite for selecting groups was that each group had a different lifestyle, educational background, and attitude toward nature. University students with an ecological background of study are supposed to be more educated on environmental questions than the general public (Pawlowski 1996). The other two groups are likely to differ in the way the forest is used. Tourists are perhaps more pro-cultural forest services than urban dwellers (Liu et al. 2021). Contrarily, ecosystem services in the urban green space are critical for urban inhabitation sustainability, thus it is important to know the demand for ecosystem services for future planning of urban green infrastructure (Wolch et al. 2014).

Groups of respondents

- *University students in environmentally-oriented fields*
- *General public (tourists)*
- *General public (urban dwellers)*

The Chinese language version of the questionnaire was distributed among the students of the Faculty of Forestry and Horticulture Sciences at Huazhong Agricultural University in Wuhan, China (*n* = 202). The Czech language questionnaires were distributed among students of the Faculty of Forestry and Wood Sciences and the Faculty of Environmental Sciences at the Czech University of Life Sciences in Prague (*n* = 213). Both language versions were distributed among students of both universities from October to November 2019. In the Czech Republic, an online version of the questionnaire was created on the LimeSurvey platform (LimeSurvey Project Team 2019). The link to fill in the questionnaire was sent by e-mail directly to the students. The online version of the Chinese questionnaire was distributed via WeChat mobile application based on the Questionnaire Star platform (Questionnaire Star Project Team 2019). Contrastingly, the general public (tourists) were interviewed in attractive natural areas. Interviewing of the general public was carried out during summer 2020, in the Czech Republic, in the National Nature Reserve of Voděradské bučiny (*n* = 100). Data collection in China was carried out in Hunan province, in the Zhangjiajie National Forest Park (*n* = 124) by personal interviews, and the subsequent completion of the questionnaire was by scanning a unique QR code on their smartphones. Selected locations in both countries are attractive tourist areas. The third surveyed group consisted of urban dwellers. The interviewing took place in the city parks of Wuhan in Hubei Province (*n* = 107), and Prague (*n* = 100). Both cities are considered metropolises in relation to the size of each country. Data collected from three different groups of individual countries allow us to compare attitudes and differences in the demand for FES across selected groups and countries.

Data collection among the urban dwellers and tourists in China applied the QR code generated from the online questionnaire. The Chinese public respondents were asked to fill out the questionnaire directly in the field by scanning the QR code on their smartphones. In the Czech Republic, the data were collected directly in the field by paper-form questionnaires. The respondents filled out their answers in approximately 8 min.

### Questionnaire structure

The first part (A) of the questionnaire focuses on the demand of FES. In closed questions, the respondents replied to each statement using the Likert scale (Likert 1932), of which we assigned the points from 1 (one) to 4 (four), with 0 as “neutral answer.” Detail definitions of the points are as follows:

- very important: (4)
- quite important: (3)
- rather unimportant: (2)
- not important at all: (1)
- I don’t know: (0)

Part A were designed into 14 statements to cover all categories of FES. The first 3 questions are linked to provisioning services, the following 6 questions reflected regulation services, and the last 5 questions represented cultural services.

The second part of the questionnaire (B) contained questions and graphic section about the expectations of forest visitors to which elements in the forest, from their point of view, influence the attractiveness of the forest. In all the 21 statements, the Likert 7-point scale (1 = least expected, 7 = most expected) was used to assess which elements in the forest from their point of view influence the attractiveness of the forest. Additionally the graphic section with 3 questions about the visual form of forest was included.

For the graphic section, modified pictures from the study by Giergiczny et al. (2015) were used. The question on the respondent’s preference of forest type included the following forest types: coniferous, deciduous, and mixed forest. This determination is related to the number of tree species, i.e., a coniferous forest was always composed of one species only. Mixed forest could be composed of 2 to 5 species, and broadleaved could be composed of 1 to 4 species. The second graphic question dealt with forest accessibility (passability of the forest) in three levels of shrub layer density. In the third part of the graphic section, the amount of larger standing and fallen pieces of natural deadwood in the forest were compared. In order to find out if respondents are aware off deadwood importance in the forest ecosystem (Bauhus et al. 2018).

Self-involvement in solving forest management questions and activities in the forest was formulated in the questions in part (C). Two categories were created on a 5-point Likert scale. The first, “Prevalence of low score,” includes answers 1–2, and the second, “Prevalence of high score” includes a range of 3–4 points. The answer, “I don’t know,” was excluded. The aim was to determine the degree of willingness to engage in or influence the forest management. The questions were divided into two groups according to their engagement: active, or passive way of participating in forest-related activities.

In the last part (D), the socio-demographic features of respondents were collected, i.e., age, gender, education. The group of students reported in addition the field of study.

### Data analyses

#### Statistical analyses

Descriptive data for the general characteristics of the respondents were presented by absolute numbers and their proportions. A group comparison of traits of the general characteristics was made via a chi-square test or the Fischer exact test for categorical data. Scores of expectation and evaluation of the FES between countries were analyzed using the Mann-Whitney test.

To identify potential predictors of the expectation and evaluation of the forest ecosystem services, binary logistic regression with a forward stepwise approach was applied. The following independent predictors associated with the dependent variables were included in the initial model: age group, gender, education level, country, and type of respondent. The statistical significance in all analyses was designated by a p-value of less than 0.05. Statistical analysis was performed using IBM SPSS statistics version 25 (IBM Corp., Armonk, NY, USA). The following independent predictors associated with the dependent variables were included in the initial model: age group (1 = the investigated age group), gender (1 = male), education level (1 = the investigated level of education), country (1 = Czech Republic) and type respondents (1 = the investigated respondent’s group) (Table A.1).

**Table 1:** Socio-demographics characteristics

Respondents	CZE *n* = 413		CHN *n* = 433		Total *n* = 846	
	n	%	n	%	n	%
Female	222	53.75	237	54.73	459	54.26
Male	191	46.25	196	45.27	387	45.74
Young adults						
18–24	169	40.92	257	59.35	426	50.35
25–34	140	33.90	116	26.79	256	30.26
Middle-aged adults						
35–44	44	10.65	46	10.62	90	10.64
45–54	22	5.33	10	2.31	32	3.78
Older adults						
55–64	19	4.60	3	0.69	22	2.60
65–79	16	3.87	1	0.23	17	2.01
80–100	3	0.73	0	0.00	3	0.35
Primary education	12	2.91	9	2.08	21	2.48
Secondary education	324	78.45	221	51.04	545	64.42
Tertiary education	77	18.64	203	46.88	280	33.10
Students	213	51.57	202	46.65	415	49.05
Tourists	100	24.21	124	28.64	224	26.48
Urban dwellers	100	24.21	107	24.71	207	24.47

## Results

### Part (D) Respondents characteristic features

Table 2 lists the contents of the respondents’ socio-demographic features: the span of respondents in the Czech Republic spread along all of the age groups, while in China, the group of oldest people (80–100 years) is not represented. Female respondents slightly prevail in both countries. The most frequently represented age group in the Czech Republic was 18–24 (40.92%), as well as in China (50.35%). Contrarily, with increasing age, the groups are less represented. In terms of education, the most numerous group is secondary education for both countries. However, there were more university graduates (tertiary education) among Chinese respondents than in the Czech Republic. In the three monitored groups, the most represented one is students, while the other two groups are evenly represented. The distribution of the number of respondents into the groups is balanced within each of the monitored countries.

**Table 2:** Evaluation of ecosystem services

Provisioning services	Mean countries			Mean students			Mean urban dwellers			Mean tourists		
	CZE	CHN	Significance	CZE	CHN	Significance	CZE	CHN	Significance	CZE	CHN	Significance
Production of oxygen and ability of trees to purify the air	3.9	3.95	x	3.92	3.93	x	3.87	3.93	x	3.88	4.00	+ + +
Wood production	3.05	3.21	+ +	3.14	3.25	x	2.81	3.16	+ +	3.11	3.20	x
Mushroom picking												
	3.23	3.06	+ +	3.08	2.92	+	3.48	3.10	+ + +	3.30	3.26	x
Regulation services												
Water retention function	3.92	3.86	+	3.96	3.85	+ +	3.86	3.87	x	3.88	3.89	x
Protection of floods												
	3.58	3.82	+ + +	3.74	3.80	x	3.29	3.82	+ + +	3.55	3.85	+ + +
Mitigation of climate change and carbon sequestration by trees												
	3.61	3.90	+ + +	3.71	3.88	+ +	3.56	3.93	+ + +	3.44	3.90	+ + +
Prevention of soil erosion												
	3.59	3.91	+ + +	3.74	3.89	+	3.37	3.93	+ + +	3.48	3.93	+ + +
Reduction of dust and noise pollution	3.23	3.69	+ + +	3.25	3.57	+ + +	3.03	3.79	+ + +	3.38	3.80	+ + +
Natural habitat for game	3.84	3.90	x	3.89	3.88	x	3.72	3.92	+	3.86	3.93	x
Cultural services												
Employment opportunities	2.74	3.10	+ + +	2.79	2.95	x	2.71	3.13	+ + +	2.64	3.31	+ + +
Public space for recreational activities	2.85	3.00	+ +	2.77	2.85	x	2.88	3.08	x	3.00	3.15	+
Provision for sports activities	2.18	2.75	+ + +	1.90	2.48	+ + +	2.32	2.81	+ +	2.65	3.13	+ + +
Enhancement for the beauty of the landscape	3.19	3.48	+ + +	3.14	3.34	+	3.11	3.51	+ +	3.37	3.69	+ + +
Cultural and spiritual importance	2.9	3.33	+ + +	2.82	3.12	+ +	3.03	3.38	+ +	2.92	3.61	+ + +
Meditation and relaxation	3.15	3.28	x	3.03	3.14	x	3.30	3.18	+	3.25	3.60	+ + +

+  +  + *p* < 0.001; +  + *p* < 0.01 + *p* < 0.05; *x* > 0.05 retain the null hypothesis

### Part (A) importance of FES and products

Respondents rated their perception of importance of fifteen FES based on a five-point Likert scale including zero. Mean scores are derived from raw data and significances are based on a non-parametric test of p-value. Mann–Whitney test was used for analyzing the non-categorical and not normally distributed data.

As part of provisioning services, oxygen production is considered equally important in all groups, the lowest rating reported from tourists. In the case of tourists, it is given slightly more importance in China. Greater importance was reported by Czech respondents to mushroom picking. In comparison, students in environmental fields of study are in consensus on the importance of all provisioning services. The least value of importance is reported by the production of wood among the Czech urban dwellers. However, provisioning services are considered very important in all groups.

Initially, it must be emphasized that all regulation FES were perceived as important in all groups in both countries. Water retention was perceived more important by Czech than Chinese. Respondents with primary education evaluated water retention with a lower score. In contrast, flood protection, mitigation of climate, and prevention of soil erosion were perceived as more important in China in all groups. Interestingly, the reduction of dust and noise pollution is perceived as more important in all groups of respondents in China than in the Czech Republic. The forest as a natural habitat for a wild game is considered on the similar level of importance across all respondents except urban dwellers respondents, of which the Czech urban dwellers perceived it significantly less important than those in China.

Within forest cultural services and groups, considerably significant responses were recorded. The employment opportunities that the forest provides are considered more important in China. In terms of individual groups, students have similar assessments in both countries. Compared to the Czech respondents, the Chinese valued recreation functions a bit more. Surprisingly, no significant differences were recorded between the respondents in the tourist group. According to the results, the Chinese are generally more aware of the importance of the forest for sports activities. The biggest drop was recorded in the student group where Czech students consider this function rather unimportant. Respondents in China, especially tourists, have a greater feeling for the forest as an element that enhances the beauty of the landscape, but this element is generally considered quite important. The cultural and spiritual importance of forests for people is more important for Chinese respondents, especially for tourists. Meditation and relaxation were generally perceived as rather important; only in the group of Chinese tourists, did the respondents lean toward very important.

The whole group of young adults, regardless of nationality, reported a high score in evaluation of employment (mean: 2.94) compared to middle and older adults (both 2.81). The cultural services were assessed as more important by higher educated respondents (mean: 3.38) than primary (3.05) and high school (2.98).

### Part (B) Expectations of forest visitors

Expectations from the forest from part (B) showed diverse results (Table 3). The demand for diverse features is a significant factor in most statements. Overall, the respondents from the Czech Republic, contrary to the Chinese respondents, expected less human encroachment in the forest, such as kiosks, parking places, sports facilities, paved paths, touristic equipment, and other attractions. In addition, Czech respondents across all groups scored high in the option of mushroom picking. In the student group, a high significant difference was reported in most options. For example, the open access to all parts of the forests was significantly more expected by the Czech than the Chinese student group. Paved paths for easy accessibility are not high scores in the Czech student group, unlike the Chinese one.

**Table 3:** Expectation from the forest

	Mean countries			Mean students			Mean Urban dwellers			Mean tourist		
	CZE	CHN	Significance	CZE	CHN	Significance	CZE	CHN	Significance	CZE	CHN	Significance
Forest with clearly visible tourist trails with signs and information boards	3.77	5.36	+ + +	3.2	5.2	+ + +	3.93	5.41	+ + +	4.82	5.56	+ + +
Forest kiosks with refreshments at the borders and the main entrances	2.79	4.85	+ + +	3.53	4.85	+ + +	1.89	4.84	+ + +	2.13	4.87	+ + +
Forest with easy accessibility for strollers and people with limited mobility (paved paths)	2.58	5.13	+ + +	1.82	5.03	+ + +	2.74	4.93	+ + +	4.03	5.46	+ + +
Forest with a bike trails network	3.98	4.76	+ + +	4.28	4.84	+ +	3.13	4.85	+ + +	4.2	4.54	x
Forest with sports facilities for active leisure time (tree climbing sites, tree climbing, zip lines, forest gyms, etc.)	3.21	4.42	+ + +	3.15	4.24	+ + +	2.84	4.44	+ + +	3.7	4.7	+ +
Forest with education trails, shelters, springs, lookout towers, etc	2.7	5.36	+ + +	1.53	5.44	+ + +	3.73	5.14	+ + +	4.17	5.4	+ + +
Forest with parking located nearby	2.6	4.51	+ + +	2.49	4.26	+ + +	2.32	4.73	+ + +	3.17	4.74	+ + +
Forest with springs, streams and lakes	4	5.89	+ + +	2.34	6.15	+ + +	5.55	5.52	x	6	5.79	+ +
Forest with wild animal observation sites	3.69	5.41	+ + +	1.86	5.44	+ + +	5.51	5.21	x	5.78	5.52	+ + +
Forest with rare plants and animal species	4.27	5.44	+ + +	3.4	5.56	+ + +	5.53	5.2	x	4.86	5.45	x
Forest with interesting natural attractions such as rocks, caves, lakes, and waterfalls	3.54	5.67	+ + +	1.74	5.94	+ + +	5.78	5.25	x	5.12	5.6	+ + +
Forest with low concentrations of trees and plants that produce allergy-causing pollen	4.44	4.66	x	5.79	4.68	+ + +	2.9	4.39	+ + +	3.11	4.84	+ +
A silent and unoccupied forest place with no disturbances	5.78	5.16	+ + +	5.77	5.32	+ +	5.59	4.91	+ +	6	5.14	+ + +
Possibility to collect mushrooms and berries	5.51	4.74	+ + +	5.4	4.81	+ + +	5.39	4.44	+ + +	5.84	4.9	+
Open access to all parts of the forest	5.26	3.94	+ + +	5.3	3.43	+ + +	5.03	3.99	+ + +	5.39	4.72	+ +
Trails and paths not overgrown by brambles and weeds	3.46	4.59	+ + +	2.71	4.33	+ + +	4.11	4.59	x	4.43	5.01	x
Hunters should control the population of wild boars and other animals (prevent their overpopulation and damage to the forest)	4.75	4.59	x	5.1	4.5	+ +	4.42	4.64	x	4.35	4.7	+ +
Clear forest land among the trees without harvest residues	4.34	5.21	+ + +	4.54	5.17	+ +	3.88	5.18	+ + +	4.37	5.31	x
Natural forest without any human interventions with impenetrable places and an oasis of calm for animals	4.82	5.42	+ + +	4.47	5.43	+ + +	5.17	5.23	x	5.19	5.57	+ + +
Breathtaking views	5.66	5.63	x	6.55	5.8	+ + +	4.86	5.32	+ +	4.58	5.64	+ + +
Romantic landscape scenery	5.69	5.51	x	6.31	5.65	+ + +	4.95	5.25	x	5.12	5.51	+ + ;
Breathing fresh air	6.06	5.87	x	5.59	6.05	+ + +	6.59	5.49	+ + +	6.55	5.9	x

+  +  + *p* < 0.001; +  + *p* < 0.01 + *p* < 0.05; *x* > 0.05 retain the null hypothesis

The bike trails, as well as parking places, are more expected by Chinese urban dwellers, while the Czech urban dwellers did not expect parking places close to the forest. Another interesting result is that Chinese urban dwellers are probably more sensitive to plants that cause allergies and thus would prefer to avoid allergy pollen plants and trees in the forest, while Czech urban dwellers are more indifferent to this. Related to the whole country sample, the urban dwellers in the Czech Republic expect more elements in the form of rocks, lakes or waterfalls and would also like to observe wildlife. Overall, all respondents’ highest expectation was for the possibility to breathe fresh air and enjoy the natural forest with a minimum of tourist attractions and other equipment. As expected, the tourist group proved significant in the international comparison, and at the same time, exhibited higher values of expectations than for the other groups, in that they expect marked hiking trails and attractive places with bodies of water in the forests.

Chinese tourists also wish to experience the undisturbed forest, but at the same time their expectations are more variable. Highest value was reported for springs, streams and lakes but also demand parking places, tourist trails and boards. Paved paths were also expected, but more from the Chinese group of tourists. Czech tourists, unlike Chinese tourists, are not very enthusiastic about placing kiosks in the forests.

In terms of education and age, respondents with primary education reported high scores on the presence of kiosks, contrary to a low score given by the older adults. Higher educated respondents rated the presence of education trails with a low score. Regarding the low occurrence of pollens, the group of young adult respondents reported a higher expectation score. Higher educated visitors preferred the harvest residues be removed from the forests.

Overall, the respondents’ highest expectation was for the possibility to breathe fresh air and enjoy the natural forest without encroachment, which is contradicted by the fact that some respondents expect a forest with recreational facilities and other tourist attractions.

### Part (B) Graphic part—Expectations of forest visitors

**Fig. 3:**
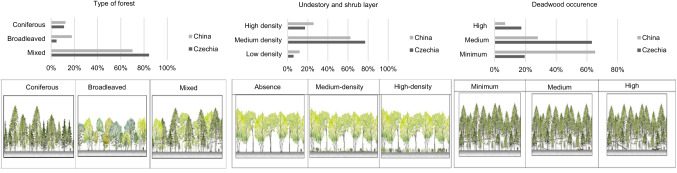
Cross-country comparisons of respondents’ preferences to the type of forest and elements in the forest

In the Graphic section of part (B), participants expressed their expectation of coniferous, broadleaved, or mixed forests (Fig. 3). The most favored was a mixed forest in both countries. The coniferous forest was the least popular in China, while contrarily, it was pure broadleaved forests in the Czech Republic. A medium amount of deadwood in the forest was the preferred choice of Czech participants, with the highest score recorded in the student group, while Chinese participants favored a minimum occurrence of deadwood in the forest across all of the groups. The Chinese, as well as the Czech participants, have a prevalent tendency to favor medium density of the understory and shrub layer. The results of expectation from part A was in consensus with findings in this part. High school and higher educated respondents expect a minimum of deadwood in the forest.

### Part (C) Participation in forest-related activities

**Fig. 4:**
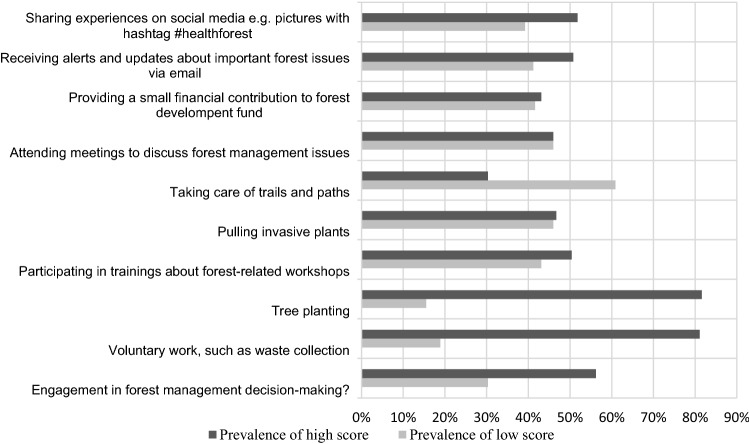
Willingness to join forest-related activities in the Czech Republic in general

**Fig. 5:**
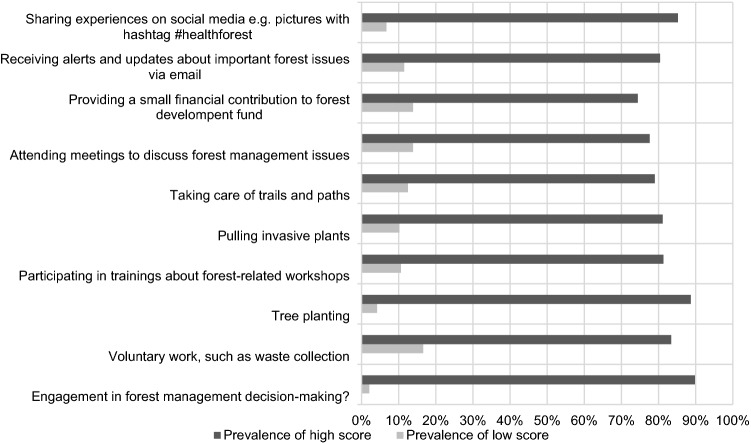
Willingness to join forest-related activities in China in general

Self-involvement was divided into groups of active and passive activities. The results of activities are shown in Fig. 4 (Czech respondents) and Fig. 5 (Chinese respondents). In general, the respondents in the Czech Republic would be willing to plant trees (81.1%) and collect waste (81.6%), while the least attractive activity for them is the maintenance of forest trails and paths (30.3%). Regarding involvement in forest-related decision-making activities, the willingness to participate was high (56.2%). From the group’s view (Fig. 4), the most willing to participate in activities were students, the weak point was shown mainly in the maintenance of trails and paths (32.4%). Besides waste collection (76%) and tree planting (76%), tourists are not overly disposed to participate in other activities. Surprisingly, the maintenance of trails and paths showed a very low level of interest (34%). Notably, disagreement was also expressed at attending forestry meetings (35%). The same is true for the urban dwellers, with the difference in that their willingness to pay a small financial contribution to forest development is greater (47%) than that of the tourists (29%).

**Fig. 6:**
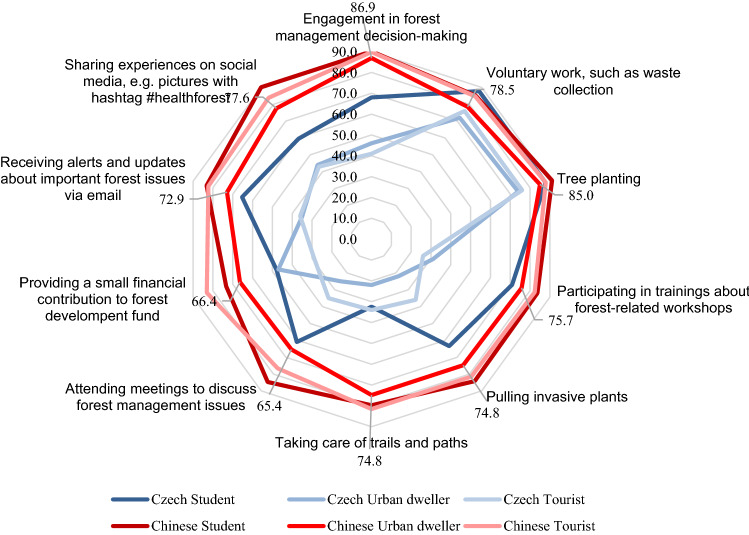
Scoring of Willingness in participation of respondents group to forest-related activities

Chinese respondents showed a strong tendency to engage in all named activities, with participating in decision-making (89.8%), tree planting (88.7%) and sharing experiences via social networks (85.2%) proving to be the most attractive. Donations were the least favored, but were still a high share of preferred activity (74.4%). The differences between the groups of respondents were not significant, and the willingness to participate prevailed in all activities (Fig. 6).

Interestingly, in this section, it showed that the male group chose rather low scores when evaluating their willingness to participate, specifically toward waste collection, trail maintenance, financial contributions, and sharing experiences through social media.

## Discussion

This research provides extensive evidence on visitors’ demand for FES and their expectations from the forest across the two investigated countries.

Concerning the first part (A) of our research, we found that the most important demand is the production of oxygen. This result ties in well with previous studies wherein the climate and oxygen-related ES were highly valuated (Gouwakinnou et al. 2019; Malik et al. 2021; Martín-López et al. 2012). The Czech students in environmental fields of study showed greater demand for regulation services than other groups, but interestingly, Chinese students did not have a significantly higher demand for FES in comparison with other groups. Although some Chinese studies reported that higher education has a positive impact on environmental awareness (Lee and Tilbury 1998; Li 2018; Yanhua 2002). Xiong et al. (2013) examined the “greener” education through academic curriculums in China higher education system and found that although the two fields of study "forestry" and "agriculture" content more environmental education elements the rest of the institutions of higher education needs to make their curriculum more "greener." Environmental education awareness development is a desirable tool for cope with the rising pressure of environmental issues that come with the rapid economy development in China. Higher interest in environmental issues among students of environmental fields was proven by Torkar and Krašovec (2019). From the results, it is clear that the provisioning and regulation services are perceived as more important than cultural services. Similar results were reported in other studies (Moutouama et al. 2019; Torkar and Krašovec 2019). When comparing our results to those of older studies carried out by Lindemann-Matthies et al. (2013), this statement is also confirmed. Cultural services were slightly more important for the tourists in both countries, especially in the valuation of forest enhancement of the beauty of the landscape, sports activities, and space for recreation. The differences due to geographical location and cultural background were obvious. Meditation, cultural-spiritual importance, and sports activities are higher valued by Chinese respondents, while collecting of mushrooms is more important for the Czechs. This is also in line with the Czech cultural tradition of mushroom picking (Šišák et al. 2016; Ministry of Agriculture 2019; Wolfslehner et al. 2019; Purwestri et al. 2020; Riedl et al. 2020) as well as traditional Chinese exercise, which has a deep tradition and makes a connection between humans and nature (Jiang and Zou 2013; Guo et al. 2014; Wang et al. 2021). Geographical location contributes to the distribution of sandstorm and industrial conditions, which also bring dust and pollution issues to the fore in China more than in the Czech Republic (Xin-fa et al. 2001; Xing et al. 2018). However, in line with the ideas of Dou et al. (2020), it can be concluded that the forest cultural services are directly influenced by cultural habits. Recent studies suggest that the perception varies depending on the type of ecosystem analyzed and socio-cultural conditions in the research area. Casado-Arzuaga et al. (2013) studied peri-urban green areas and their respondents were more likely to report that cultural services are the most important. Contrarily, in Northern Kenya, the most important features were the provisioning services (Caroline et al. 2018), which was also the case in both the Vietnam Bach ma National Park (Hong and Saizen 2019), and West Java, Indonesia (Muhamad et al. 2014).

Based on the investigation of Chinese visitors’ expectations, the obvious conclusion is that the existence of various kinds of recreational facilities make their stay in nature more comfortable and play a key role in their expectations of the forest, especially with urban dwellers and tourists. Although at the same time, they demand a natural and silent forest without any human interventions. Similar results were found by De Meo et al. (2015). It reflects the idealized image of the forest – “how it should look like, according to the conservation of natural values,” regardless of the visitors’ needs. In recent decades, the growing population and increasing standard of living resulted in higher demand for nature tourism in China (LI Shi-dong and CHEN Xin-feng 2007; National Bureau of Statistics of China 2019). The number of visitors to National Forest parks and demand for forest recreation in China is continuously growing. It gives rise to building new recreational facilities in the parks such as hotels, parking places, etc., that threaten the environment (Wang et al. 2012; Chen and Nakama 2013). The sum of these findings exerts pressure to generate measures for sustainable management and recreational spatial planning more than ever before (Oku and Fukamachi 2006; Chen and Nakama 2013). Contrarily, in the Czech Republic, forest visitors prefer the forest without additional facilities. Similar outcomes were found in the study of Drábková and Šišák (2013), where forest visitors predominantly demand maintained paths. Other studies from the urban green spaces have shown a higher preference for gravel surface and less maintained trails when compared to asphalt, but it also depends on the type and age of the respondents (Arnberger et al. 2010; Reichhart and Arnberger 2010). On the other hand, the establishment of new recreational facilities attracts visitors to the less appealing areas and thus reduces the pressure in overcrowded green areas. In addition, well-placed signage of paths and bike trails may prevent conflict between different groups of tourists (Arnberger et al. 2018). Urban dwellers in both countries seem to be more tolerant of allergy trees and plants than other groups. However, the sensitivity for pollen in the forest among urban dwellers group was recorded more among Chinese. Cheng et al. (2015) found that one of the main trees that caused allergies in Wuhan is richly planted Platanus. Spring and autumn are the most pollen abundant seasons in Wuhan. The volume of allergenic pollen in the atmosphere is dependent upon the climate, topography, and vegetation (D’Amato et al. 2007; Cheng et al. 2015). Greater interest in wildlife observation among Czech urban dwellers compare to whole country sample may stem from a lack of contact with wildlife in cities. The daily contact with nature over recent decades decline (Kay et al. 2022). Nevertheless, the interactions with wildlife can be good for human health and wellbeing (Cox and Gaston 2018). These expectations can be beneficial for the development of citizen science (Lee et al. 2020; Franco and Cappa 2021).

Historically, mixed forests occur naturally in the Czech Republic (Filkova et al. 2014). Nevertheless, in the past two centuries, forests were transformed by human intervention into species compositions of forests in which coniferous trees dominate that do not correspond to the prescribed target species composition (Tomášková 2004). A high proportion of Norway spruce (Picea abies), simultaneously with climate change, have negatively affected the state of forests and gave rise to the bark beetle calamity. An ongoing bark beetle outbreak causing mass deforestation is turning Czech forests from carbon sinks into significant sources of greenhouse gas emissions. Thus, the bark beetle outbreak negatively influenced the regulation services (Hlásny et al. 2019). One of the recommended solutions is the restoration of mixed forests, which is currently publicly supported (Hynek 1997; Tomášková, 2004; Seidl et al. 2014; Ministry of Agriculture 2019). It seems that the higher demand for mixed forests reflects the current situation. This is partially confirmed by Price (2003) who claims that the preference of coniferous is not due to the choice of tree species per se, but their use in intensive forest management or geometric designs in the landscape that are judged visually poor according to recreational potential (Price 2003). Similar results were obtained across Europe (Edwards et al. 2012; Lindemann-Matthies et al. 2013; De Meo et al. 2015; Gerstenberg and Hofmann 2016; Grilli et al. 2016). Moreover, the review study of Gundersen and Frivold (2008) of Scandinavian quantitative studies concludes that people´s preferences for tree species depend on the package of other visual factors, as well as what kind of forest people are used to. The southern part of China belongs to the low latitude region, and due to the unique geographical location, climatic conditions, and complex topography, broadleaved forests account for the largest area among the different forest types, while the area of coniferous is relatively low (Chen Jianwei 2015). Therefore, considering the distribution of tree species and personal preferences, the government and people in the South prefer broadleaved forest species.

Our findings illustrate an interesting difference in perceiving the occurrence of decayed and deadwood lying on the ground. While Czech respondents preferred its medium occurrence, the higher preference was recorded by the Czech student group. The higher level of education affected the inclination to appreciate the amount of deadwood in the forest, which was confirmed in previous studies as well (Tyrväinen et al. 2001; Rathmann et al. 2020). It seems that educated people are more aware of forest biodiversity. However, in China, the preference for minimum deadwood prevails. This is similar to previous studies. In the Finland studies of Tyrväinen et al. (2001, 2003) the visitors to the forest do not prefer deadwood in the forest due to decrease the scenic value, limited sight and accessibility. Other studies also indicate visitors prefer a low amount of deadwood and favor clear visibility and security (Tyrväinen et al. 2001; Edwards et al. 2012; Arnberger et al. 2018). Thus, cultural and geographical differences are important elements in different perceptions of decayed wood. The key is the educational background of the public, concerning the ecological benefits and importance of decaying wood for the forest. The study area also probably plays an important role, e.g., in mountain forests in Italy and Sarajevo, as tourists reported a higher preference for the occurrence of deadwood (Pastorella et al. 2016). Our study areas included mainly recreational forests. Across all groups of respondents, a moderate understorey and shrub layer was considered to be most attractive. People in Europe generally prefer good visibility in the forest (Edwards et al. 2012).

The most popular forest-related self-involved activity across the countries studied is clearly tree planting. The findings are directly in line with Šišák (2011), who found that tree planting is considered the most important forest operation by forest visitors. Although tree planting can contribute to ecological and social well-being, it is necessary to consider this effort as one component of multifaceted solutions to environmental problems. The complexity in planning and following long-term monitoring of planted trees must be taken into account in cooperation with stakeholders (Brancalion and Holl 2020). Different factors might relate to the willingness of the community to participate in the tree-planting program. For instance, economic benefit (Filius, 1997; Khuc et al. 2021) and environmental protection (soil erosion reduction, water, and biodiversity conservation) are reasons (Filius, 1997; Villamayor-Tomas et al. 2019) supporting sustainable forest management (Ansong and Røskaft 2014), or as part of the climate change mitigation (Acquah and Onumah 2011) were reported by the community as reasons to participate in such a program. Men were more reticent than women in assessing their willingness to engage in activities. This corresponds to Wilson’s finding (2000) that women are more likely to volunteer than men. The fact that the Chinese are willing to use social networks by sharing activities in connection with the forest may correspond to the high number and rapid growth of social media users in China (64.6%) (Kemp 2021). Although the number of Czech social media users is also quite high (56.3%), they were more conservative in willing to share forest-related content (Czech Statistical Office, 2021).

## Conclusion

While previous studies have mostly looked at the demand for FES in one country, our study addressed forest perceptions and the demand for FES within two countries. Based on the results, it was found that the most valuable FES in both countries is oxygen production and the ability of forests to purify the air. In general, the demand for provisioning and regulation services is higher than for cultural services. Differences were noted between some responses of different social groups: students, urban dwellers, and tourists, which illustrate their profiles. Czech students with an environmental focus of study demonstrated a higher environmental education background in some points, while Chinese students were at the level of other groups. Unsurprisingly, tourists showed higher demand for recreational facilities in the forest, but generally, the Chinese respondents had a higher demand for recreational facilities and services in the forest compared to the Czech ones. This is alarming given the emerging pressure on FES as it needs to be taken into account in forest planning and management to meet the objectives of sustainable forest management and forest protection. Differences arising from the geographical and cultural bases were also evident, especially in the different demands for cultural services and regulation services. Chinese respondents demand the cultural, spiritual, and meditation services associated with their culture, while the Czech respondents highly valued mushroom picking. The reduction of noise and dust pollution is demanded more by Chinese respondents as expected, due to a higher level of air pollution from industry and sandstorms. However, the positive finding is that people are generally aware of the value of FES, as none of the important FES were assessed negatively by the respondents. The Chinese respondents were more inclined toward voluntary forestry-related activities in all respects. The popularity of tree-planting is common to both nations. The Chinese, unlike the Czechs, showed a willingness to share their experience of the forest through social networks. These findings on the voluntary participation of forest visitors can be used in the future to involve the public in forest-related activities and thus, strengthen public awareness about the FES and encourage individuals in nature conservation activities.

## Data Availability

(data transparency) Not applicable.

## Appendix

See Tables 4 and 5.

**Table 4:** Significant preferences in each category

	Country	Type of respondent	Age	Education level	Gender
Evaluation					
Wood		(−) Students*			
Mushroom	(−) Czech**	(+) Students*			
Water retention				(+) Primary education*	
Flood protection	(+) Czech*	(−) Students*			
Carbon sequestration		(−) Students*	(+) Older adults*	(−) Tertiary education*	
Soil erosion		(+) Urban dwellers*			
Dust/noise	(+) Czech**	(+) Urban dwellers**	(−) Older adults*	(+) Secondary education*	
Employment			(−) Young adults*	(+) Secondary education**	
Recreation		(+) Students*			
Sports	(+) Czech**	(+) Students**			
(+) Tourists**					
Landscape	(+) Czech**	(−) Tourists*			
Culture	(+) Czech*			(−) Tertiary education*	
Meditation	(+) Czech*	(+) Students*			
Forest presence of services expectation	Country	Type of respondent	Age	Education level	Gender
Kiosks	(+) Czech**	(−) Students**	(+) Older adults*	(−) Primary education*	
Access for strollers	(+) Czech**	(−) Tourists**			
Bike trails	(+) Czech**	(−) Students*			
Leisure facilities	(+) Czech**	(−) Tourists*			
Education trails	(+) Czech**	(+) Students**		(+) Tertiary education*	
Parking	(+) Czech**	(−) Students*			
Spring streams	(+) Czech**	(+) Students**		(+) Tertiary education**	
Animal observation	(+) Czech**	(+) Students**		(−) Secondary education*	
Rare species	(+) Czech**	(+) Students**			
Natural attractions	(+) Czech**	(+) Students**			
(−) Urban dwellers*		(+) Tertiary education*			
Low pollen		(−) Students**	(−) Young adults*		
Silence	(−) Czech**				
Game control		(−) Students*			
Remove residue of harvest	(+) Czech**			(+) Tertiary education**	(−) Male*
Nature of forest	(+) Czech*	(−) Tourists*			
Breathtaking view		(−) Students**	(+) Young adults*	(+) Secondary education*	
Romantic scenery		(−) Students**			
Pictures	Country	Type of respondent	Age	Education level	Gender
Mixed forest	(+) Czech**	(−) Urban dwellers*	(−) Young adults*		
Broadleaves	(−) Czech**	(+) Urban dwellers*			
Low-density	(−) Czech*				
Medium-density	(+) Czech**				
High-density	(−) Czech*				
Minimum deadwood	(−) Czech**	(−) Students*	(+) Older adults*	(−) Tertiary education**	(−) Male*
Medium deadwood	(+) Czech**		(+) Young adults**		(+) Male*
High deadwood	(+) Czech**		(+) Middle-aged adults*		
Action					
Waste collection	(+) Czech*		(+) Older adults**		(+) Male*
Tree planting	(+) Czech**		(+) Older adults*		
Workshop	(+) Czech**	(−) Students**	(+) Older adults*		
Invasive plant	(+) Czech**	(−) Students**			
(+) Tourists*					
Trail maintenance	(+) Czech**	(+) Urban dwellers*			(+) Male*
Meetings, discussions	(+) Czech**	(−) Students**	(−) Middle-aged adults*		
Fee contribution	(+) Czech**				(+) Male*
Email notification	(+) Czech**	(−) Students**	(+) Older adults*		
Social media	(+) Czech**		(+) Older adults**		(+) Male*

**Table 5:** Independent variables of the binary logistic regression analysis

Independent variables	Explanations
Age group of the respondents^a^	
Young adults	13–34 y
Middle age adults	35–54 y
Older adults	above 55 y
Gender (1 = male)	
Education level^a^	
Primary education	Primary school
Secondary education	High school, Vocational school
Higher education	University and above
Country (1 = Czech Republic)	
Type of respondents^a^	
Students	
Tourists	
City dwellers	

^*^ the investigated variable was coded as 1 (one), the rest of the groups were coded as 0 (zero)
